# Supplementary material for: Modeling glioblastoma heterogeneity as a dynamic network of cell states
Source: Mol Syst Biol. 2021 Sep 16;17(9):e10105. doi: 10.15252/msb.202010105 (PMC8444284; doi:10.15252/msb.202010105)
Supplement: Supplementary file 6 — Source Data for Figure 5 [file MSB-17-e10105-s004.zip › Figure5A_sourcedata/GSEA_3017/nosigdb_stateB.GseaPreranked.1622795021725/RICHARDS_ZHONG_NATURE_2018_NPCS_UPREG.html]

Details for gene set RICHARDS\_ZHONG\_NATURE\_2018\_NPCS\_UPREG[GSEA]

|  || Dataset | state43017 |
| Phenotype | NoPhenotypeAvailable |
| Upregulated in class | na\_pos |
| GeneSet | RICHARDS\_ZHONG\_NATURE\_2018\_NPCS\_UPREG |
| Enrichment Score (ES) | 0.6667629 |
| Normalized Enrichment Score (NES) | 3.8244126 |
| Nominal p-value | 0.0 |
| FDR q-value | 0.0 |
| FWER p-Value | 0.0 |
Table: GSEA Results Summary

  

Fig 1: Enrichment plot: RICHARDS\_ZHONG\_NATURE\_2018\_NPCS\_UPREG      
 Profile of the Running ES Score & Positions of GeneSet Members on the Rank Ordered List

  

| PROBE | GENE SYMBOL | GENE\_TITLE | RANK IN GENE LIST | RANK METRIC SCORE | RUNNING ES | CORE ENRICHMENT || 1 | HIST1H4C |  |  | 0 | 1.373 | 0.0278 | Yes |
| 2 | TOP2A |  |  | 3 | 1.079 | 0.0466 | Yes |
| 3 | RRM2 |  |  | 7 | 0.875 | 0.0599 | Yes |
| 4 | UBE2C |  |  | 11 | 0.802 | 0.0716 | Yes |
| 5 | TPX2 |  |  | 12 | 0.801 | 0.0878 | Yes |
| 6 | NUSAP1 |  |  | 13 | 0.801 | 0.1040 | Yes |
| 7 | HMGB2 |  |  | 14 | 0.786 | 0.1199 | Yes |
| 8 | FBXO5 |  |  | 15 | 0.785 | 0.1358 | Yes |
| 9 | SPC25 |  |  | 17 | 0.763 | 0.1498 | Yes |
| 10 | PRC1 |  |  | 18 | 0.756 | 0.1651 | Yes |
| 11 | TACC3 |  |  | 21 | 0.741 | 0.1771 | Yes |
| 12 | GTSE1 |  |  | 22 | 0.732 | 0.1919 | Yes |
| 13 | HJURP |  |  | 23 | 0.731 | 0.2067 | Yes |
| 14 | KIF23 |  |  | 24 | 0.721 | 0.2213 | Yes |
| 15 | ASPM |  |  | 26 | 0.695 | 0.2338 | Yes |
| 16 | NDC80 |  |  | 27 | 0.695 | 0.2479 | Yes |
| 17 | ATAD2 |  |  | 28 | 0.685 | 0.2618 | Yes |
| 18 | KIF15 |  |  | 29 | 0.684 | 0.2756 | Yes |
| 19 | KPNA2 |  |  | 33 | 0.676 | 0.2848 | Yes |
| 20 | CENPF |  |  | 34 | 0.670 | 0.2984 | Yes |
| 21 | SMC4 |  |  | 41 | 0.655 | 0.3026 | Yes |
| 22 | MELK |  |  | 42 | 0.655 | 0.3159 | Yes |
| 23 | NUF2 |  |  | 44 | 0.651 | 0.3276 | Yes |
| 24 | PRR11 |  |  | 45 | 0.649 | 0.3407 | Yes |
| 25 | DLGAP5 |  |  | 46 | 0.647 | 0.3538 | Yes |
| 26 | DHFR |  |  | 49 | 0.638 | 0.3637 | Yes |
| 27 | CDCA5 |  |  | 52 | 0.632 | 0.3735 | Yes |
| 28 | CKAP2 |  |  | 53 | 0.628 | 0.3862 | Yes |
| 29 | CCNA2 |  |  | 54 | 0.626 | 0.3989 | Yes |
| 30 | CKAP2L |  |  | 59 | 0.611 | 0.4053 | Yes |
| 31 | CENPU |  |  | 60 | 0.605 | 0.4176 | Yes |
| 32 | TAGLN2 |  |  | 61 | 0.604 | 0.4298 | Yes |
| 33 | CENPK |  |  | 62 | 0.603 | 0.4420 | Yes |
| 34 | KIF2C |  |  | 66 | 0.591 | 0.4494 | Yes |
| 35 | SMC2 |  |  | 68 | 0.586 | 0.4598 | Yes |
| 36 | CDK1 |  |  | 69 | 0.585 | 0.4716 | Yes |
| 37 | H2AFX |  |  | 71 | 0.576 | 0.4818 | Yes |
| 38 | CKS1B |  |  | 72 | 0.575 | 0.4934 | Yes |
| 39 | MAD2L1 |  |  | 77 | 0.569 | 0.4989 | Yes |
| 40 | CKS2 |  |  | 83 | 0.558 | 0.5028 | Yes |
| 41 | AURKB |  |  | 89 | 0.548 | 0.5064 | Yes |
| 42 | CENPM |  |  | 92 | 0.544 | 0.5144 | Yes |
| 43 | CCNB1 |  |  | 93 | 0.544 | 0.5254 | Yes |
| 44 | PBK |  |  | 95 | 0.540 | 0.5348 | Yes |
| 45 | RRM1 |  |  | 103 | 0.520 | 0.5349 | Yes |
| 46 | CDKN3 |  |  | 108 | 0.513 | 0.5393 | Yes |
| 47 | UBE2T |  |  | 110 | 0.512 | 0.5481 | Yes |
| 48 | CDCA3 |  |  | 111 | 0.511 | 0.5585 | Yes |
| 49 | BUB1 |  |  | 113 | 0.508 | 0.5673 | Yes |
| 50 | TUBB4B |  |  | 114 | 0.507 | 0.5775 | Yes |
| 51 | BIRC5 |  |  | 116 | 0.504 | 0.5862 | Yes |
| 52 | CLSPN |  |  | 117 | 0.503 | 0.5964 | Yes |
| 53 | DTYMK |  |  | 118 | 0.503 | 0.6066 | Yes |
| 54 | DUT |  |  | 124 | 0.494 | 0.6091 | Yes |
| 55 | TMPO |  |  | 127 | 0.492 | 0.6161 | Yes |
| 56 | ITGB3BP |  |  | 128 | 0.490 | 0.6260 | Yes |
| 57 | GMNN |  |  | 135 | 0.484 | 0.6268 | Yes |
| 58 | C21orf58 |  |  | 142 | 0.470 | 0.6273 | Yes |
| 59 | CDCA4 |  |  | 148 | 0.461 | 0.6292 | Yes |
| 60 | ORC6 |  |  | 150 | 0.461 | 0.6370 | Yes |
| 61 | KIF22 |  |  | 152 | 0.459 | 0.6448 | Yes |
| 62 | KNSTRN |  |  | 155 | 0.456 | 0.6511 | Yes |
| 63 | SPAG5 |  |  | 156 | 0.455 | 0.6603 | Yes |
| 64 | DEPDC1B |  |  | 177 | 0.436 | 0.6392 | Yes |
| 65 | MKI67 |  |  | 179 | 0.436 | 0.6465 | Yes |
| 66 | FEN1 |  |  | 180 | 0.435 | 0.6553 | Yes |
| 67 | CDC20 |  |  | 184 | 0.431 | 0.6596 | Yes |
| 68 | MIS18BP1 |  |  | 186 | 0.430 | 0.6668 | Yes |
| 69 | MCM4 |  |  | 199 | 0.418 | 0.6573 | No |
| 70 | ZWINT |  |  | 212 | 0.407 | 0.6476 | No |
| 71 | H2AFZ |  |  | 216 | 0.405 | 0.6513 | No |
| 72 | PCNA |  |  | 242 | 0.386 | 0.6217 | No |
| 73 | HMGN2 |  |  | 243 | 0.386 | 0.6295 | No |
| 74 | CCNB2 |  |  | 250 | 0.379 | 0.6282 | No |
| 75 | CENPW |  |  | 267 | 0.366 | 0.6117 | No |
| 76 | ANP32E |  |  | 280 | 0.358 | 0.6010 | No |
| 77 | CENPN |  |  | 309 | 0.343 | 0.5661 | No |
| 78 | OIP5 |  |  | 315 | 0.341 | 0.5655 | No |
| 79 | BTG3 |  |  | 337 | 0.331 | 0.5408 | No |
| 80 | PHGDH |  |  | 344 | 0.328 | 0.5385 | No |
| 81 | HAT1 |  |  | 352 | 0.324 | 0.5346 | No |
| 82 | WDR34 |  |  | 366 | 0.321 | 0.5217 | No |
| 83 | PTTG1 |  |  | 413 | 0.302 | 0.4590 | No |
| 84 | RNASEH2A |  |  | 478 | 0.285 | 0.3691 | No |
| 85 | APOLD1 |  |  | 487 | 0.284 | 0.3629 | No |
| 86 | RPA3 |  |  | 584 | 0.267 | 0.2248 | No |
| 87 | PAX6 |  |  | 617 | 0.261 | 0.1823 | No |
| 88 | UHRF1 |  |  | 652 | 0.255 | 0.1366 | No |
| 89 | VIM |  |  | 692 | -0.267 | 0.0837 | No |
| 90 | ID4 |  |  | 708 | -0.285 | 0.0671 | No |
| 91 | HSPB1 |  |  | 730 | -0.378 | 0.0433 | No |
Table: GSEA details [plain text format]

  

Fig 2: RICHARDS\_ZHONG\_NATURE\_2018\_NPCS\_UPREG: Random ES distribution      
 Gene set null distribution of ES for **RICHARDS\_ZHONG\_NATURE\_2018\_NPCS\_UPREG**
